# Hypoxia‐guided treatment planning for lung cancer with dose painting by numbers

**DOI:** 10.1002/acm2.14609

**Published:** 2024-12-20

**Authors:** Yazhou Li, Yuanyuan Ma, Jieyan Wu, Hui Zhang, Hongyi Cai, Xinguo Liu, Qiang Li

**Affiliations:** ^1^ Institute of Modern Physics Chinese Academy of Sciences Lanzhou China; ^2^ Key Laboratory of Heavy Ion Radiation Biology and Medicine of Chinese Academy of Sciences Lanzhou China; ^3^ Key Laboratory of Basic Research on Heavy Ion Radiation Application in Medicine Lanzhou Gansu Province China; ^4^ University of Chinese Academy of Sciences Beijing China; ^5^ Gansu Provincial Hospital Lanzhou China

**Keywords:** dose painting by numbers, hypoxia, lung cancer, normal tissue complication probability, tumor control probability

## Abstract

Tumor hypoxia significantly impacts the efficacy of radiotherapy. Recent developments in the technique of dose painting by numbers (DPBN) promise to improve the tumor control probability (TCP) in conventional radiotherapy for hypoxic cancer. The study initially combined the DPBN method with hypoxia‐guided dose distribution optimization to overcome hypoxia for lung cancers and evaluated the effectiveness and appropriateness for clinical use of the DPBN plans. ^18^F‐FMISO PET‐CT scans from 13 lung cancer patients were retrospectively employed in our study to make hypoxia‐guided radiotherapy. In the clinic, TCP and normal tissue complication probability (NTCP) derived from the DPBN plans in comparison to conventional intensity modulated radiation therapy (IMRT) plans were evaluated. Additionally, in order to investigate the improved clinical suitability, the robustness of DPBN plans in response to potential patient positioning errors and radiation resistance variations throughout the treatment course was assessed. The DPBN approach, employing voxelized prescription doses, led to an average increase of 24.47% in TCP, alongside a reduction of 1.83% in NTCP, compared to the conventional radiotherapy treatment plans. Regarding the robustness of the DPBN plans, it was observed that positional uncertainties were limited to 2 mm and radiosensitivity deviations were within 4%. The lung NTCP showed a 0.05% increase when the isocenter was moved by 3 mm in any direction, suggesting that the DPBN plan meets clinical acceptability criteria. Our study has shown that the DPBN technique has significant potential as an innovative approach to enhance the efficacy of radiotherapy for lung cancer with hypoxic regions.

## 1 INTRODUCTION

Tumor hypoxia, which is prevalent in solid tumors, significantly undermines the efficacy of radiotherapy. The prior observations by Gray et al. in 1953 first illuminated the radiobiological significance of hypoxia. Subsequent research on malignant cell cultures emphasized that cancer cells demonstrate an increased resistance to ionizing radiation when in a hypoxic state.^1^ Hence, the implementation of hypoxia‐focused radiotherapy for providing better treatment outcomes is a crucial therapeutic strategy.

Various approaches have been investigated, including the technique of dose painting. The idea of dose painting, initially proposed by Ling et al.,^2^ has been extensively explored as a means to selectively escalate the dose in areas exhibiting increased radio‐resistance. Initially, dose painting was applied through the uniform boosting of tumor sub‐volumes, a method referred to as dose painting by contour (DPBC).^2^, ^3^ Although DPBC has the potential to counteract hypoxia and improve local control, the indiscriminate escalation of dose across the entire gross tumor volume (GTV) could result in detrimental toxicities due to excessive exposure of adjacent normal tissues.^4^, ^5^

In order to address these constraints, Alber et al. and Bentzen introduced the innovative approach of dose painting by numbers (DPBN),^6^, ^7^ which advocates for variable dose delivery based on the specific functional characteristics of tumor tissues. DPBN customizes dose prescriptions at the voxel level, informed by functional imaging,^7^, ^8^ allowing for precise dose distributions within the tumor. Previous studies have indicated that integrating functional imaging data into intensity modulated radiation therapy (IMRT) optimization algorithms through DPBN enhances the treatment efficacy by enabling more precise dose distributions.^8^, ^9^, ^10^

Up to now, despite DPBN's potential to pursue tumor hypoxia effectively, most planning studies conducted with DPBN involved head and neck cancers.^4^ This is, however, a limitation when considering the implementation of lung cancer. Only one related method was found for DPBN used in patients with lung cancer.^11^ The study has focused on optimizing the expected tumor control probability (TCP) for the DPBN plan to achieve robustness for TCP and associated dose volume parameters for organs at risk (OARs). To our knowledge, no relevant study has yet integrated DPBN with hypoxia‐guided dose distribution optimization for assessing its clinical feasibility in lung cancer patients.

Therefore, the purpose of our study was to introduce a DPBN optimization framework customized for lung cancer treatment. Our objectives involved optimizing key objective functions in radiotherapy treatment plans, such as maximizing the target volume receiving doses above a specific threshold while minimizing the maximum dose to critical organs. In order to show the clinical effectiveness, we assessed the TCP and normal tissue complication probability (NTCP) derived from the DPBN plans in contrast to conventional IMRT plans. Additionally, in order to investigate the improved clinical suitability, we assessed the robustness of the DPBN plans against potential patient positioning errors and radiation resistance variations throughout the treatment course.

## 2 METHODS AND MATERIALS

### 2.1 Patient data

In this study, ^18^F‐FMISO PET‐CT datasets for 13 lung cancer patients were acquired from an institutional review board‐approved retrospective data collection protocol. These PET‐CT datasets were included in this study under the IRB‐approved protocol by the Academic Committee of the Institute of Modern Physics, Chinese Academy of Sciences (No. 2023‐16). The PET images were reconstructed with a resolution of 4.07 mm × 4.07 mm × 2 mm, whereas the CT images had a resolution of 0.97 mm × 0.97 mm × 5 mm. To align with the PET image spatial resolution, the CT images were downsampled. PET uptake segmentation was confined to values within the CT‐based planning gross tumor volume (PGTV). The dimensions of images were standardized after PET and CT image registration (MIM Software Inc., USA). These PET and CT scans served as the basis for radiotherapy treatment planning, with critical structures delineated accordingly.

Utilizing the Eclipse software version 13.6 (Varian Medical Systems, USA), an experienced physician delineated the GTV. The PGTV was created by extending the GTV by 3 mm in all directions. The delineation of OAR was facilitated by automated sketching tools with the MANTEIA software (MANTEIA Software Inc., China).

The hypoxia tumor volume (HTV) was defined as the sub‐volume within the GTV exhibiting a tumor to muscle ratio (T/M) ≥ 1.5 on the ^18^F‐FMISO PET scans.^12^ The subscapular muscle was used as the reference in this context. The threshold of 1.5 as the cutoff for hypoxic regions was applied within the GTV using a sub‐thresholding algorithm in the Eclipse software (version 13.6). Table 1 summarizes the patient and tumor characteristics of the study cohort.

**TABLE 1:** Patient characteristics.

Patient	Age	Sex	Site	PGTV volume (cm^3^)	GTV volume (cm^3^)	HTV volume (cm^3^)
1	47	F	Right upper	147.3	99.5	8.6
2	62	M	Left lower	74.8	56.7	32.7
3	61	M	Right hilar	60.5	37.4	7.7
4	75	M	Right lower	198.6	139.3	48.6
5	71	M	Right hilar	148.5	93.9	34.2
6	76	M	Left hilar	212.6	126.4	49.7
7	56	M	Right hilar	76.4	44.3	10.6
8	73	M	Left upper	51.3	26.4	7.3
9	67	M	Right upper	123.2	69	29.1
10	69	M	Right hilar	181.1	116	10.3
11	61	F	Left upper	25.3	12	2.9
12	68	M	Right hilar	59.1	34	3.5
13	46	F	Right upper	116	68.7	18.5

Abbreviations: GTV, gross tumor volume; HTV, hypoxia tumor volume; PGTV, planning gross tumor volume.

### 2.2 Radiotherapy treatment planning

Two separate treatment plans were created for every patient using the matRad open‐source radiotherapy treatment planning system,^13^ employing identical beam angle settings: a conventional IMRT plan and a DPBN plan targeting the HTV with voxel‐specific prescription doses.

The optimization process involves minimizing a cost function, a method inspired by the principles of IMRT. The cost function for optimizing the planning target volume (PTV) is defined as the quadratic sum of the discrepancies between the calculated and prescribed doses:

(1) $$F_{\mathrm{PTV}}\;\left(\vec{N}\right)=\sum_{i\in \mathrm{PTV}}w_{\mathrm{PTV}}{\left(D_{act.}^{i}\left(\vec{N}\right)-D_{\mathrm{presc}.}\right)}^{2}$$

where *w*_PTV_ is the weighting factor, having a significant impact on guiding the result of the optimization, $D_{act.}^{j}\left(\vec{N}\right)$ is the actual dose of voxel *i*, and $D_{\mathrm{presc}.}$ is the dose to the tumor as prescribed by a physician.

In the optimization function, the $D_{\mathrm{presc}.}$ is a uniform prescription dose to the target volume for the conventional IMRT plan. Conversely, the $D_{\mathrm{presc}.}$ is a heterogeneous prescription dose to a voxel in the target volume for the DPBN plan. The $D_{\mathrm{presc}.}$ is different for each voxel within the HTV and it can be provided by a function related to radiosensitivity voxel by voxel.

The conventional IMRT plan, referred to as the standard (STD) plan, specified a uniform prescription dose of 60 Gy across 30 fractions directed at the PGTV. Conversely, the DPBN plan assigned individualized doses to each voxel within the HTV as dictated by Equation (7), while maintaining a 60 Gy dose for the remaining PGTV area, excluding the HTV. Both plan types adhered to identical normal tissue constraints to ensure consistency in comparison.

### 2.3 Radiosensitivity quantification

Tumor hypoxia imaging via ^18^F‐FMISO, a kind of nitroimidazole tracer, takes advantage of its specific retention mechanism caused by the suppression of nitroaromatic enzymatic reduction in oxygen‐rich conditions.^14^ This different uptake, relying on local tissue oxygen levels, clearly distinguishes areas of the tumor with different signal strengths and outlines regions with low oxygen levels from those with normal oxygen levels. The analysis employed 18F‐FMISO PET imaging to assess the different radiation resistance within the tumor microenvironment, highlighting hypoxic regions as exhibiting heightened radiotherapy resistance.^15^, ^16^

The process of translating PET image intensities to radiation sensitivities encompassed two crucial steps. Initially, a conversion function that translates the PET signal intensities into oxygen partial pressures was established by fitting tracer uptake data to a model (Equation 2) as below:

(2) $$\mathrm{Uptake}=A-\frac{B\times pO_{2}}{C+pO_{2}}$$

where *pO*_2_ is the local oxygen tension. *A*, *B*, and *C* are fitted parameters. *A* = 10.9, *B* = 10.7, *C* = 2.5.^17^ Experimental data on ^18^F‐FMISO uptake has been obtained from Jason et al.^18^

Then, the alteration of radioresistance in response to local oxygen levels (called oxygen enhancement ratio, OER) is modeled by Equation (3)^10^, ^19^ as follows:

(3) $$\mathrm{OER}=\frac{\mathrm{OE}R_{max}\times \left(k+pO_{2}\right)}{k+\mathrm{OE}R_{max}\times pO_{2}}$$

here, OER*_max_* represents the peak effect observed in the absence of oxygen, *k* is a reaction constant, and *pO_2_* denotes the local oxygen tension. OER*_max_* = 3, *k* = 2.5 mmHg.^20^, ^21^ It is worth noting that while some studies may refer to this concept as the hypoxia reduction factor (HRF) or dose modification factors (*f*),^22^ to maintain clarity and consistency, we used OER throughout this paper. Additionally, Table 2 provides a detailed view of the OER values for all the patients under investigation.

**TABLE 2:** The OER values of the patients in this study group.

Patients ID	1	2	3	4	5	6	7	8	9	10	11	12	13
Max(OER)	1.2	1.17	1.11	1.22	1.25	1.25	1.09	1.25	1.46	1.07	1.49	1.13	1.17
Mean(OER)	1.13	1.11	1.09	1.11	1.15	1.13	1.06	1.19	1.17	1.04	1.33	1.1	1.11

Abbreviations: OER, oxygen enhancement ratio.

### 2.4 Prescription dose

A potential approach for dose prescription function could be given by the following expression^23^:

(4) $$D\left(r\right)=n\frac{\alpha \left(r\right)}{\beta \left(r\right)}\left[\sqrt{1+\frac{1}{n}\frac{4\beta \left(r\right)}{\alpha^{2}\left(r\right)}\ln\left(\frac{Vn_{0}\left(r\right)}{-\ln P}\right)}-1\right]$$

(5) $$\propto \left(r\right)=\frac{\alpha_{oxic}}{OER\left(r\right)}$$

(6) $$\beta \;\left(r\right)=\frac{\beta_{oxic}}{OER{\left(r\right)}^{2}}$$

where *n* is the number of treatment fractions, α(r) and β(r) reflect radiosensitivity parameters adjusted for local oxygen levels via the OER, *n_0_(r)* signifies the initial cell density distribution and is assumed to be 10^8^ cells here, *V* represents the tumor volume, while *P* stands for the local TCP, set at 95% for this analysis.

In situations where tumors are delineated into individual voxels using contemporary imaging techniques, the discrete units are accommodated by the analytic model shown in Equation (4). The prescription dose on each voxel, with its specific volume *V_i_*, is considered in Equation (7) as follows:

(7) $$D\;\left(V_{i}\right)=n\frac{\alpha \left(V_{i}\right)}{\beta \left(V_{i}\right)}\left[\sqrt{1+\frac{1}{n}\frac{4\beta \left(V_{i}\right)}{\alpha^{2}\left(V_{i}\right)}\ln\left(\frac{Vn_{0}\left(V_{i}\right)}{-\ln P}\right)}-1\right]$$

### 2.5 Plan evaluation

To assess and compare treatment plans effectively, we utilized the TCP and NTCP metrics. These metrics are crucial in the evaluation process as they offer insights into the efficacy and safety of radiation therapy plans. The accurate calculation of TCP relies on a thorough comprehension of the heterogeneous biological distributions within tumor regions. Specifically, TCP is derived using the following formula^24^, ^25^:

(8) $$\mathrm{TC}P_{i}=\exp\left(-\exp\left(\ln N_{i}-\alpha_{i}nd_{i}-\beta_{i}nd_{i}^{2}\right)\right)$$

(9) $$\mathrm{TCP}=\prod_{i}\mathrm{TC}P_{i}$$

Here, $N_{i}$ represents the initial count of clonogenic cells within a specific tumor voxel. The parameters $\alpha_{i}$ and $\beta_{i}$ correspond to the linear and quadratic coefficients of cellular radiosensitivity respectively. The coefficients are crucial because they differ between the hypoxic and aerobic regions within the tumor. For hypoxic regions, $\alpha_{i}$ and $\beta_{i}$ can be determined from Equations (5) and (6). On the other hand, in areas with oxygen, these coefficients are $\alpha_{oxic}$ and $\beta_{oxic}$. Furthermore, *n* indicates the number of radiation dose fractions.

The assessment of NTCP is conducted via the Lyman‐Kutcher‐Burman model, as delineated in the references.^26^, ^27^, ^28^

(10) $$\mathrm{NTCP}=\frac{1}{\sqrt{2\pi }}\;\int_{-\infty }^{t}e^{-\frac{x^{2}}{2}}dx$$

(11) $$t=\frac{\mathrm{EUD}-TD_{50}}{m\;TD_{50}}$$

(12) $$\mathrm{EUD}={\left(\sum_{i}v_{i}D_{i}^{\frac{1}{n}}\right)}^{n}$$

In this study, the following parameters were used: $n=0.87,m=0.18,\;TD_{50}=24.5Gy,\;\alpha /\beta =3Gy$ for the lungs, $n=0.05,m=0.175,\;TD_{50}=66.5Gy,\;\alpha /\beta =10Gy$ for the spinal cord, $n=0.06,m=0.11,\;TD_{50}=68Gy,\;\alpha /\beta =10Gy$ for the esophagus, $n=0.35,m=0.10,\;TD_{50}=48Gy,\;\alpha /\beta =10Gy$ for the heart.^28^

To assess the robustness of the DPBN plans, we incorporated simulated errors in positioning during patient treatment. The errors in patient positioning were investigated by simulating a rigid shift of the patient relative to the isocenter. Additionally, we implemented a method to artificially augment radiation resistance factor values by a 1% gradient across each voxel. This approach allows us to estimate the plan's robustness against variations in radiation resistance throughout the course of radiotherapy.

The comparison between TCP and NTCP parameters was conducted using the Wilcoxon signed‐rank sum test, using the STD plan as the standard for comparison. A *p*‐value less than 0.05 in a two‐tailed test was considered to indicate statistical significance. The SPSS software was employed for statistical analyses, ensuring a comprehensive evaluation of the data.^29^

## 3 RESULTS

In our study, we selected ^18^F‐FMISO PET‐CT images from 13 patients to devise two distinct radiotherapy treatment plans per patient: the STD plan and the innovative DPBN plan. For all 26 created plans, we achieved our target volume objectives, with both STD and DPBN plans adhering to the constraints for OARs.

**FIGURE 1:**
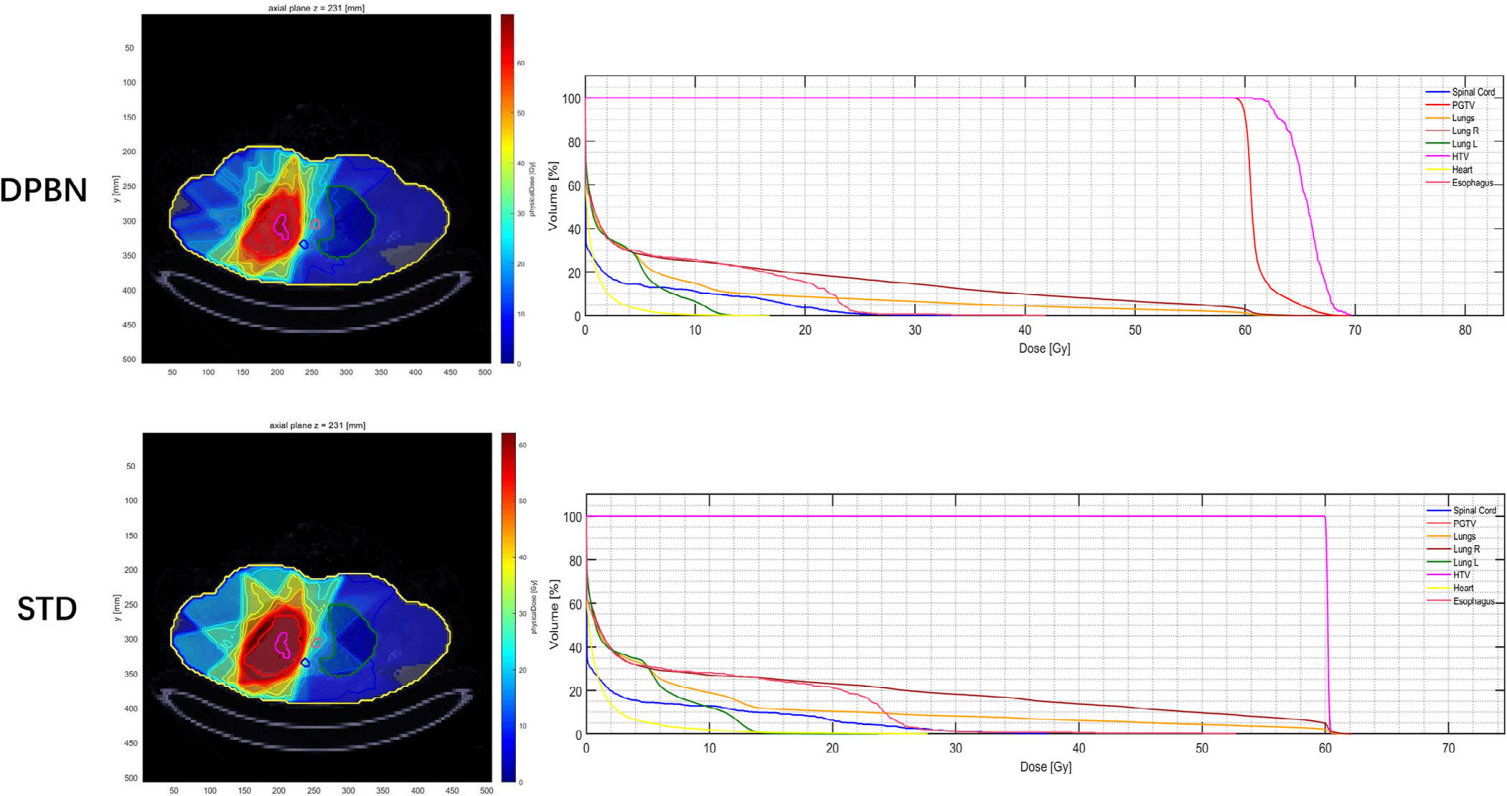
The DVH and isocenter cross‐sectional plots of one representative example are presented. The top plot representing the DPBN plan and the bottom plot representing the STD plan. DPBN, Dose painting by numbers; STD, standard

Figure 1 depicts a dose volume histogram (DVH) and isocenter cross‐sectional plot for a representative patient, comparing the dose distributions of the STD and DPBN plans. This comparison revealed a minimal discrepancy in the dose delivered to the OARs across both methodologies. However, a marked contrast is observed in the dose distribution within the target volume. Notably, the STD intensity‐modulated plan ensured homogeneous coverage of the prescribed 60 Gy dose across the PGTV and its sub‐targets (GTV and HTV). Conversely, the DPBN plan utilized a more precise approach, delivering heterogeneously higher doses than that prescribed to the HTV, showing the plan's capability for targeted dose escalation.

**FIGURE 2:**
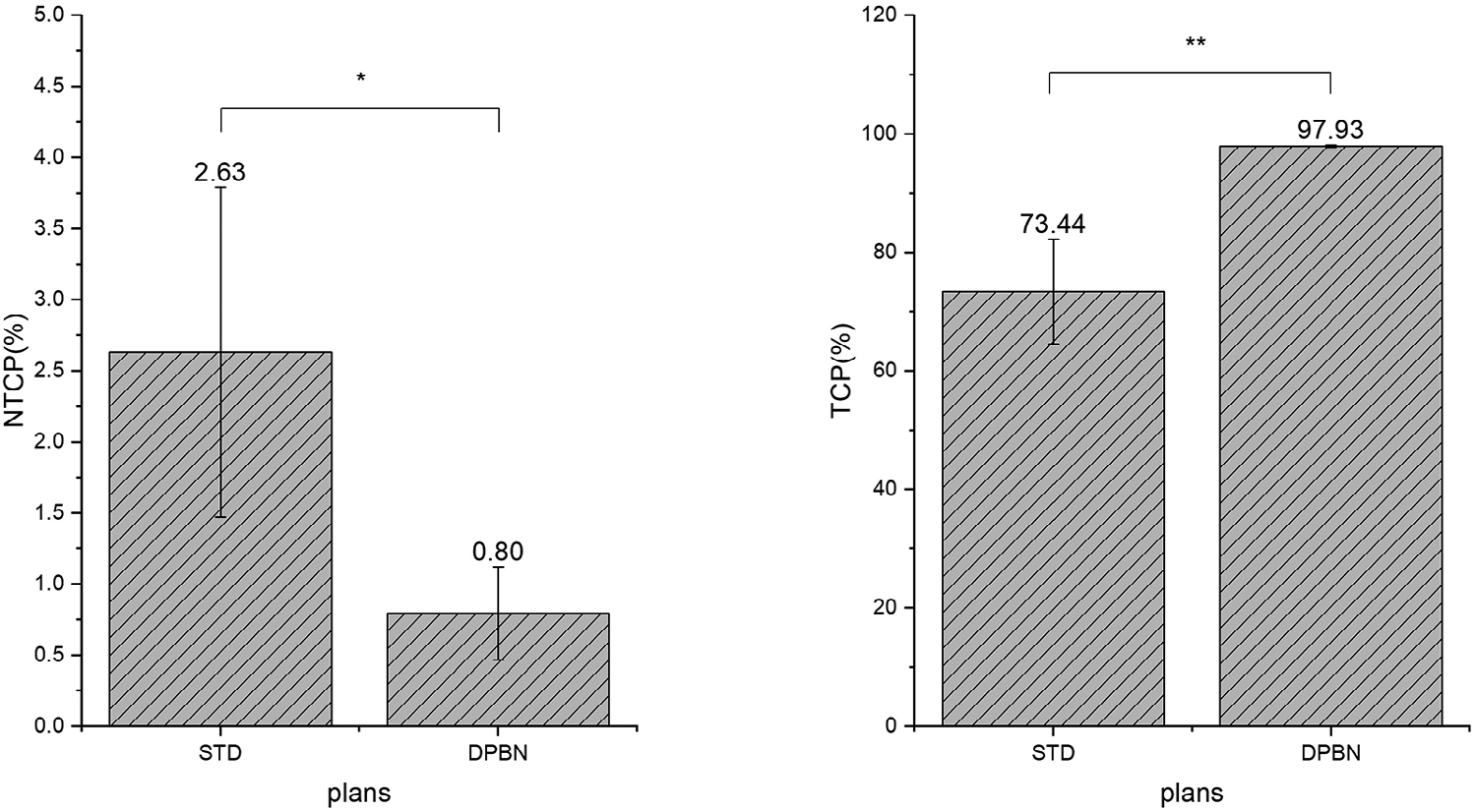
The error bars of the NTCP and TCP for the 13 patients. Means ± SEs of the original data are presented. The values above the error bars represent the mean NTCP/TCP. The symbols on the error bars are the results referring to the statistical significance analysis of the differences of the average NTCP/TCP (with the STD plan as a reference) (** *p* ≤ 0.001; * *p* ≤ 0.05; ns, *p *> 0.05). NTCP, normal tissue complication probability; SEs, standard errors; STD, standard; TCP, tumor control probability.

Figure 2 displays the averages and standard errors (SEs) of the TCP and NTCP calculated from all the plans for the 13 patients. The analysis of TCP across the cohort of 13 patients revealed mean TCPs of 73.44% for the STD plans and 97.91% for the DPBN plans. This demonstrated a substantial enhancement in efficacy with the DPBN plans, which exhibited nearly a 24.5% higher TCP compared to their STD plans. This difference between the two types of plans was found to have a statistically significant impact (*p ≤* 0.001).

For the evaluation of NTCP, we focused on the unaffected lung tissue, specifically excluding the PGTV from our assessment. Analysis of the NTCP values for lung complications across the 13 patients revealed mean percentages of 2.63% for the STD plans and 0.8% for the DPBN plans. This suggests a significant decrease of almost 1.83% in NTCP values for lung complications with the DPBN plans compared to the STD plans, a difference that was statistically significant (*p *≤ 0.05). The detailed NTCP values for lung complications are illustrated in Figure 2. The statistical findings obtained from SPSS are summarized in Table 3.

**TABLE 3:** The Means ± SEs for TCP, NTCP, and the corresponding *p*‐values of the 13 patients.

	TCP	NTCP
STD (%)	73.44 ± 8.85	2.63 ± 1.16
DPBN (%)	97.93 ± 0.20	0.80 ± 0.33
*p‐*value	0.001	0.002

Abbreviations: DPBN, Dose painting by numbers; NTCP, normal tissue complication probability; SEs, standard errors; STD, standard; TCP, tumor control probability.

Furthermore, our analysis extended to other critical organs, including the spinal cord, esophagus, and heart. No statistically significant differences in NTCP values were observed between the STD and DPBN plans for these organs, with values nearly negligible in both planning approaches. The patients' average NTCP is shown in Table 4.

**TABLE 4:** The mean NTCPs of the 13 patients for various organs.

	STD	DPBN
Spinal cord (%)	< 0.01	< 0.01
Esophagus (%)	< 0.01	< 0.01
Heart (%)	< 0.01	< 0.01

Abbreviations: DPBN, Dose painting by numbers; NTCP, Normal tissue complication probability; STD, standard.

**FIGURE 3:**
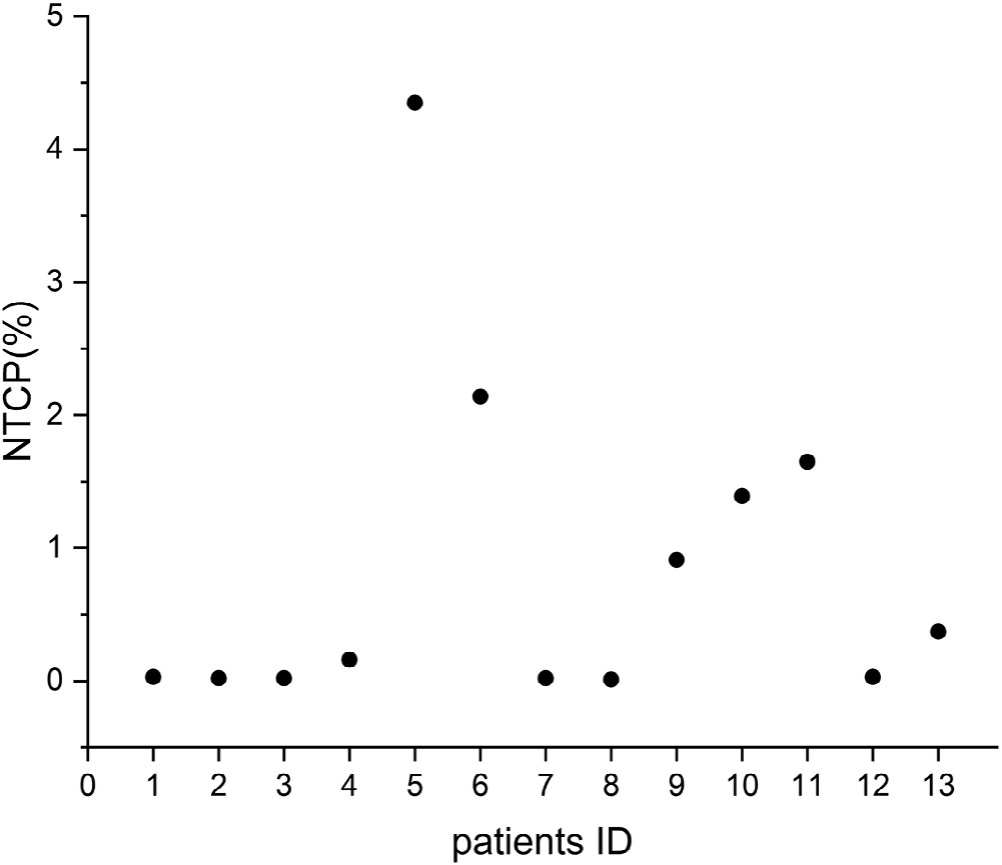
The scatter of the average NTCPs for the 13 patients. The average NTCP values were determined by recalculating NTCP after shifting the isocenter of the plans by 3 mm. NTCP, Normal tissue complication probability.

In addition to efficacy, safety considerations were crucial in our study. We assessed potential damage to lung tissue under the DPBN planning, particularly when the plan's isocenter was moved by 3 mm in any direction, which is a frequent positional error in the radiotherapy course. The results of the NTCP model indicated that such movement had a minimal impact on the risk of lung injury, with an average NTCP value of 0.85%, as shown in Figure 3. Compared with the original DPBN plans (Figure 2), the difference in NTCP value between them was 0.05%, which is insignificant and negligible. In addition, most NTCPs are less than 1%, with approximately half of them being close to zero. Patient 5 had a significantly higher NTCP value than the others, which may be due to the normal lung tissue receiving a relatively higher dose.

**FIGURE 4:**
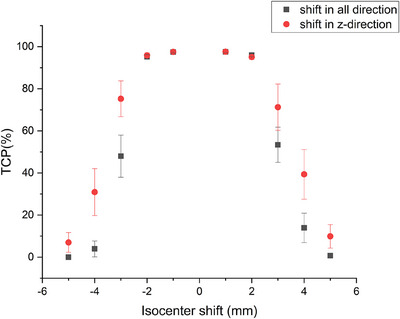
The error scatter of the average TCPs and plan isocenter shifts for the 13 patients. Means ± SEs of the original data are presented. The square points represent the isocenter shift in any direction and the circle points represent the isocenter shift in z‐direction. TCP, Tumor control probability.

We conducted a simulation to evaluate the impact of positional errors on treatment planning by recalculating the dose distribution using a method involving beam isocenter adjustment. Based on our clinical experience and observations, we found that the maximum positional error for chest tumor patients can reach up to 5 mm. Therefore, we shift the beam isocenter by increments of 1 mm, 2 mm, 3 mm, 4 mm, and 5 mm, respectively, in order to recalculate the plans’ dose distribution and reassess TCP and NTCP. This simulation aims to assess the dose distribution in the presence of different positional errors. Due to the frequent positional errors that occur in the z‐direction during the radiotherapy course, the DPBN plans were recalibrated to account for the shifts in the z‐direction, ranging from 1 mm to 5 mm for each case. Our results showed that the average TCP remained above 95% for the isocenter movements of up to 2 mm, as depicted in Figure 4. Furthermore, we extended our analysis to simulate three‐dimensional movements of the body, adjusting the plan's isocenter from 1 mm to 5 mm in all directions simultaneously. The TCP outcomes were compared with the z‐directional shifts alone. As a result, they were generally consistent. This consistency highlighted the clinical robustness of the DPBN plan, especially with the help of advancements in modern image‐guided radiotherapy technologies.

This study also explored a comprehensive assessment of the OER, a crucial radiosensitivity parameter, in the context of the DPBN plans. To scrutinize the impact of OER variations, we systematically raised the OER value by small 1% steps, ranging from 1% to 10%, across all HTV voxels. Subsequent recalculations were performed on the DPBN plans and their TCPs in order to assess the impact of these modifications.

**FIGURE 5:**
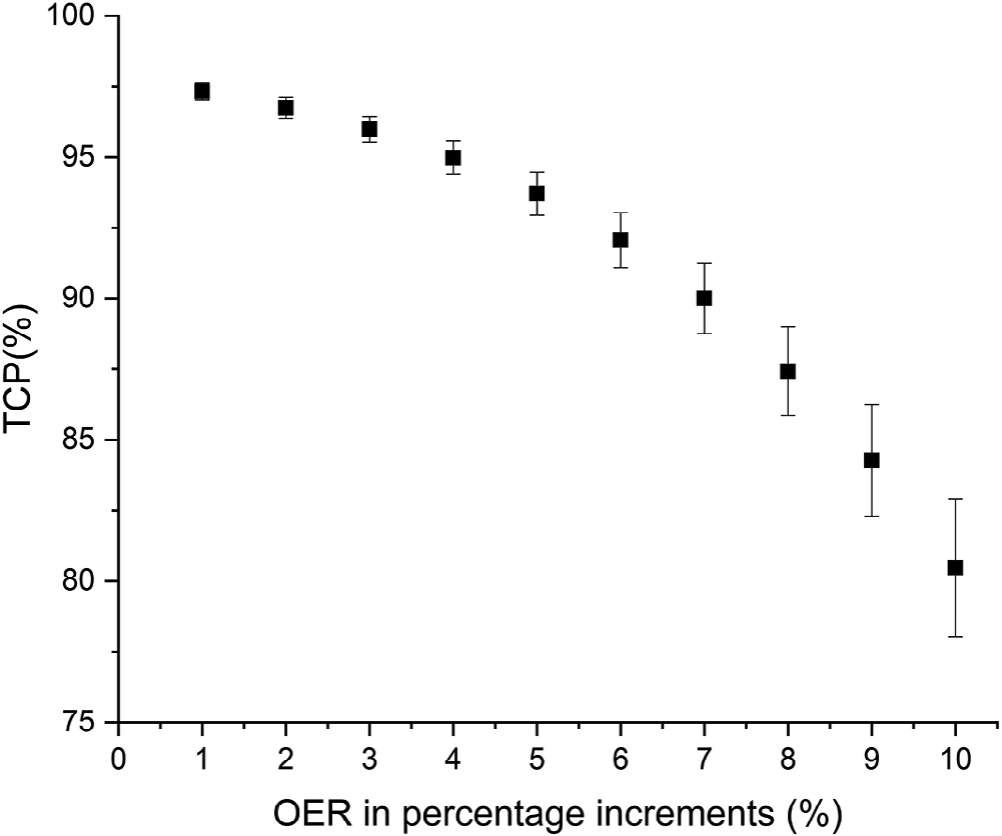
The error scatter observed in the TCP and OER increments across the 13 patients. Means ± SEs of the original data are presented. OER, oxygen enhancement ratio; SEs, standard errors; TCP, tumor control probability.

Our findings revealed that the average TCP remained above 95% when the OER increment was kept within 4%. When the OER increase extended from 5% to 7%, the average TCP remained robust, exceeding 90%. However, surpassing a 7% increase in OER value resulted in a decline of the average TCP to below 90%. These crucial data points, illustrating the TCP's sensitivity to varying OER levels in the DPBN plans, are depicted in Figure 5.

Finally, the duration of each patient under both the STD and DPBN plans was meticulously recorded. The average time for STD plans was 109.5 s, with a maximum duration of 157.7 s and a minimum duration of 57.2 s. For DPBN plans, the average time was 132.3 s, with a maximum duration of 295.1 s and a minimum duration of 52.8 s. The time required for both the STD and DPBN plans is comparable, indicating that the clinical feasibility of the DPBN plan remains unaffected by time constraints.

## 4 DISCUSSION

The efficacy of conventional radiotherapy treatment plans depends on multiple tumor‐specific factors, including tumor location, tumor volume, and the extent of hypoxia within the tumor. Among these factors, hypoxia is considered to be the most significant. Traditional approaches frequently struggle to ensure precise delivery of high doses to the vital target volumes, however, the increased dosage might not consistently deposit into the hypoxic areas due to the inherent randomness in dose distribution. In contrast, the DPBN treatment plan offers a precise solution by precisely prescribing high doses customized to the unique needs of each voxel within the hypoxic region. This method ensures a targeted high dose distribution, effectively directing the treatment against different degrees of hypoxia. This study first integrated the DPBN technique with dose distribution optimization to combat hypoxia in lung cancer treatment. Moreover, the efficacy and clinical suitability of the DPBN plan were evaluated.

Figure 2 illustrates that the TCP of the DPBN plan was higher than that of the STD plan, while the NTCP of the DPBN was lower compared to the STD plan, suggesting that, in contrast to the STD plan, the DPBN plan exhibited superior capacity in safeguarding OAR and mitigating the impact of hypoxia on the radiotherapy efficacy.

Figures 4 and 5 depict that the TCP remained consistently above 95% even in the presence of positional uncertainties up to 2 mm and radiosensitivity deviations up to 4%, thereby demonstrating the clinical suitability of the DPBN technique for lung cancer. When the positional error was 3 mm, the NTCP of the lung was 0.85%, indicating minimal impact indeed. The findings emphasized the accuracy and reliability of DPBN in adapting to inherent treatment variances. Such robustness enhances the clinical feasibility of DPBN, offering a significant advance in the targeted therapy of lung cancer.

In our study, the positron emission tomography and computed tomography (PET‐CT) images were selected over simulated CT to reduce the risk of misalignment between hypoxic regions and dose distribution, which was primarily attributed to the potential mismatch between simulated CT and PET images. Despite advances in image processing software, these misalignments have the potential to cause substantial discrepancies. Utilizing PET‐CT for both the identification of hypoxic areas and radiotherapy treatment plans sustains the accuracy of our study.

However, our study is not without its limitations. Specifically, we focused on patients with lung tumors without accounting for respiratory motion, nor did we consider variances in oxygen partial pressure over time. Future studies could benefit from dynamic PET imaging or adjusting prescribed doses based on sequential PET scans to refine the heterogeneity of dose prescriptions. Moreover, the methodology to translate tracer uptake values to oxygen partial pressures remained approximate, highlighting the need for more accurate conversion techniques. ^18^F‐FMISO is commonly used to identify hypoxic areas, but the varying effectiveness of different tracers indicates that there may be variability in detecting regions of low oxygen levels. The determination of the threshold for hypoxia also remains a topic of debate, with the T/M ratio of 1.5 commonly employed, yet other criteria exist. Lastly, the findings of this study rely solely on biological modeling. Therefore, these results should be validated through a prospective clinical trial in the future.

True hypoxia‐guided radiotherapy represents voxel by voxel targeting, where each tumor voxel is associated with a specific radiosensitivity and precise dose distributions for each voxel within the tumor. Our study primarily shows the application of DPBN in devising voxel‐specific dose prescriptions to pursue tumor hypoxia effectively using photon radiotherapy. The investigation into particle therapy, including proton and heavy ion therapies, presents an exciting avenue for future research aimed at surmounting tumor hypoxia challenges. In our next research phase, we plan to undertake comprehensive studies on particle therapy as a strategic method against hypoxia in lung cancer patients.

## 5 CONCLUSION

This retrospective study employed ^18^F‐FMISO PET‐CT images for delineating hypoxic regions in lung cancer and personalized prescription doses based on a function associated with the radiation resistance factor OER. Subsequently, through the application of the DPBN technique, we ensured precise dose modulation to the hypoxic areas. This novel DPBN method was compared with conventional radiotherapy strategy under TCP and NTCP metrics for assessment. The average increase in TCP of 24.47% alongside the decrease in NTCP of 1.83% indicates that employing the ^18^F‐FMISO PET‐guided DPBN technique is a more effective approach for lung cancer with hypoxic regions. The DPBN plan was assessed for its ability to withstand changes in patient positioning and resistance to radiation. The results indicated that even within the range of positional errors commonly observed in clinical practice, the TCP of the target volume remained at 95%, showing the efficacy of the DPBN technique. Furthermore, there was no rise in NTCP for the lung, providing additional evidence of the effectiveness and suitability of the DPBN technique in a clinical setting.

## AUTHOR CONTRIBUTIONS

All authors contributed to the study conception and design. Yazhou Li was involved in conceptualization, methodology, investigation, writing original draft, and visualization. Yuanyuan Ma was involved in methodology, reviewing and editing the original draft. Jieyan Wu was involved in data collection and analysis. Hui Zhang was involved in reviewing. Xinguo Liu was involved in reviewing and funding acquisition. Qiang Li and Hongyi Cai were involved in conceptualization, reviewing, editing the original draft, supervision, and funding acquisition. All authors read and approved the final manuscript.

## CONFLICT OF INTEREST STATEMENT

The authors declare no conflicts of interest.

## Data Availability

The data that support the findings of this study are available on request from the corresponding author. The data are not publicly available due to privacy or ethical restrictions.
